# Attention Problems Predict Risk of Violence and Rehabilitative Engagement in Mentally Disordered Offenders

**DOI:** 10.3389/fpsyt.2019.00279

**Published:** 2019-05-07

**Authors:** Ignazio Puzzo, Ottilie Sedgwick, Rachel Kelly, Ben Greer, Veena Kumari, Gisli Guðjónsson, Susan Young

**Affiliations:** ^1^Department of Psychology, City University of London, London, United Kingdom; ^2^Centre for Cognitive Neuroscience, Department of Life Sciences, Brunel University London, London, United Kingdom; ^3^Department of Psychology, Institute of Psychiatry, Psychology & Neuroscience, King’s College London, London, United Kingdom; ^4^University of Bath, Bath, United Kingdom; ^5^University of Reykjavik, Reykjavik, Iceland; ^6^Psychology Services Limited, London, United Kingdom

**Keywords:** mentally disordered offenders, attention, impulsivity, hyperactivity, risk of violence, therapeutic engagement

## Abstract

Mentally disordered offenders (MDOs) endorse difficulties with attention, impulsivity, and hyperactivity. Assessing these difficulties among MDOs may confer practical benefits for the management and provision of care for this population, by informing strategies to improve rehabilitative engagement and risk assessments for violence. However, there is a dearth of literature exploring these cognitive problems in MDOs in relation to outcome factors. Forty-eight MDOs from a high-security hospital completed the QbTest, which measures the domains of inattention, impulsivity, and hyperactivity. Comprehensive file review of clinical and occupational/vocational rehabilitative engagement and Historical Clinical Risk Management-20 (HCR-20) were used as outcome measures of interest. Participants displayed greater cognitive deficits in attention, impulsivity, and hyperactivity compared to the general population. The domain of inattention and omission errors was related to occupational/vocational therapy engagement as well as a higher risk of present and future violence as measured by the HCR-20. The findings suggest that QbTest is a helpful objective tool that could be incorporated into the assessment of MDOs. Specifically, inattention emerged as a strong predictor of patients’ risk of violence as well as patient’s vocational therapy engagement. Therefore, cognitive skills programs targeting attention problems should be introduced to improve outcomes for this population.

## Introduction

Difficulties in the domains of attention, hyperactivity, and impulsivity are present across adult forensic and forensic psychiatric populations at both clinical (i.e., attention deficit hyperactivity disorder, ADHD) and subclinical levels (1, 2). A meta-analysis by Young et al. (3) indicated that 31% of adult prisoners meet criteria for ADHD, compared to 2.5–4.2% in community adult populations. A systematic review by Fazel and Seewald (1) suggested that prisoners with a psychiatric diagnosis of schizophrenia spectrum disorder, mood disorder, or personality disorder displayed impairments in these three domains. Within UK forensic mental health services, it is estimated that 6–12.9% of adult mentally disordered offenders (MDOs) endorse significant problems in these domains, based upon diagnostic screening measures for ADHD (4).

In addition to diagnostic screening measures, MDOs have also reported significantly greater rates of impulsivity in self-report measures, relative to normative populations (5, 6). MDOs have additionally demonstrated poorer performance than healthy controls in behavioral assessments of impulsivity and attention, including continuous performance tasks (CPTs) (7–9). Research utilizing CPTs has found that the traits of impulsivity and inattention are part of the clinical presentation of mental disorders that are prevalent in forensic psychiatric populations, such as schizophrenia and antisocial personality disorder (ASPD) (10). For example, ASPD and schizophrenia are both characterized by impulsive and inattentive behavior (11).

Indeed, individuals with schizophrenia or ASPD display a greater number of commission errors, or responses to nontarget stimuli, compared to healthy controls, providing objective support for the presence of impulsive responding in this group (12). It has also been demonstrated that those with a diagnosis of ASPD or schizophrenia have a significantly higher number of omission errors (missing a target when it appears) in CPTs compared to control groups (13). As omission errors measure inattention, this suggests that those with a diagnosis of schizophrenia or ASPD display higher levels of inattentive behavior.

There are practical benefits in better understanding the relationship between difficulties in attention, hyperactivity, and impulsivity and rehabilitative engagement among MDOs. For example, if inattention, impulsivity, and hyperactivity are found to be good predictors of rehabilitative engagement, then assessing these cognitive abilities may enable the identification of patients at risk of low engagement early on in their clinical recovery journey and provide them with additional support in order to maximize rehabilitative engagement. This may in turn have a benefit in tackling initial disengagement from rehabilitative activities which may culminate in a total discontinuation of the activity, with rehabilitative program attrition rates between 37% and 50% reported among forensic inpatients (14). Attrition is likely to hamper patients’ progression through their care program and ultimate progression out of forensic mental health services. Moreover, attrition has been associated with a significantly greater risk of future reoffending compared to individuals receiving no treatment at all (15).

There are also practical benefits in better understanding the relationship between difficulties in attention, hyperactivity, and impulsivity and risk of violence (16). The prediction of violence is an integral issue within forensic settings, with violence risk assessment and management being considered key aspects of clinical practice in prison populations (17). Identifying risk factors for violence in these populations is therefore potentially life-saving, as it can allow professionals to predict violence, understand its causes, and prevent reoccurrence (18). A substantial amount of research suggests that violent offenders display impairments in controlling their behavior and impulses (19). Impulsivity and inattention are clinical features of mental disorders including ASPD and schizophrenia, which are particularly associated with a greater risk of violence and are overrepresented in these settings (19). Overall, a limited number of studies have investigated impulsivity and attention in MDOs. For example, Enticott et al. (20) investigated cognitive inhibitory control using Stroop and negative priming tasks and its association with self-reported impulsiveness among violent offenders with schizophrenia. They found that negative priming, but not the Stroop effect, was impaired among violent offenders with schizophrenia, and there was no association between reduced inhibition and self-reported impulsivity. Along similar lines, Meijers et al. (21) investigated differences in executive functions (including response inhibition, planning, attention, set shifting, working memory, and impulsivity/reward sensitivity) between violent and nonviolent offenders (but not mentally disordered). Their finding suggested that violent offenders performed significantly worse on the stop-signal task (response inhibition) compared to nonviolent offenders. Most recently, Stratton et al. (22) compared neuropsychological performance between offenders with schizophrenia who had committed homicide and nonviolent schizophrenia controls. They observed greater cognitive dysfunction on measures of executive functioning, a finding in line with earlier findings of Barkataki et al. (7) and, additionally, on measures of memory and the intellectual functioning composite score, in the violent schizophrenia group relative to the nonviolent schizophrenia comparison sample.

Overall, there are few data that have directly explored whether specific and objective measures of inattention, impulsivity, and hyperactivity are associated with risk of violence in MDOs (23), despite theoretical frameworks suggesting that impulsivity is an important construct when formulating violence risk in individuals with psychosis (24).

The current study utilized a sample of MDOs from a UK high-security hospital to explore the domains of attention, hyperactivity, and impulsivity as measured by the Quantified Behavioural Test (QbTest) (25) in relation to risk of violence and engagement in rehabilitative activities. Assessing attention, hyperactivity, and impulsivity within forensic mental health services is typically reliant upon the clinical interpretation of subjective information sources, including self- and informant-report measures, and is therefore susceptible to issues of reliability and consistency (26). The QbTest, however, offers an objective measure of these three behavioral domains, coupling an infra-red sensor with a CPT. There is no published literature regarding the use of the QbTest in a MDO sample, and the main domains (attention, hyperactivity, and impulsivity) were developed for use with patients with ADHD. Therefore, those domains were operationalized and computed by taking information from different parts (quartiles) of the computer task and have different weightings for parameters within them in order to assist with sensitivity to ADHD-type symptoms. It is therefore unknown how sensitive these domains are for the MDO population and for correlating with risk of violence or rehabilitative engagement. In light of that, the present study in addition to the test domain also explored number of omission and commission errors, which may display a rawer form of measuring inattention and impulsivity.

Although previous research studies have focused on clinical rehabilitative activities such as psychological therapy, difficulties in these domains could also be associated with other vocational therapies (27). Therefore, we reviewed clinical records to obtain a clinically informed impression of both clinical and vocational therapy engagement. As meta-analyses have identified the Historical Clinical Risk Management-20 (HCR-20) (28) as a good predictor of risk of violence in psychiatric forensic settings (29), the current study utilized this risk assessment tool.

The present study aims to answer three main questions: a) Are MDOs significantly impaired in the domains of attention, impulsivity, and hyperactivity as assessed by the QbTest compared to the normative population? b) Are the QbTest domains of inattention, impulsivity, and hyperactivity individually related to a participant’s risk of violence, as measured by the HCR-20, or rehabilitative engagement (both clinical and vocational therapies)? c) Do omission and/or commission errors relate to risk of violence or rehabilitative engagement?

Based on previous findings we hypothesized that MDOs resident at a high-security hospital would be significantly impaired in the domains of impulsivity, attention, and hyperactivity compared to the normative population, as assessed by the QbTest. We also expected to find links between domains of inattention, impulsivity, and hyperactivity as well as omission and/or commission errors and risk of violence and rehabilitative engagement.

## Methods

### Participants

Participants in this study were MDO patients recruited from a UK high-secure hospital, all of whom were adult men detained under The Mental Health Act and therefore in-patients. Fifty-five participants were initially recruited for this study, but seven participants were excluded after either withdrawing from testing, or due to an inability to follow the instructions given. The final sample size was *N* = 48, aged between 21 and 60 years (*M* = 38.8, SD = 9.6). The patient’s IQ score ranged between 73 and 115 (*M* = 93.5, SD = 12.3). Thirty-four participants were recruited from low-dependency wards (70.8%), four participants from medium-dependency wards (8.3%), one participant from a high-dependency ward (2.1%), and nine participants from admission wards (18.8%). Twenty-six participants (54.2% of the whole sample) presented with a primary schizophrenia spectrum disorder diagnosis, with eight (30%) of them also presenting with a secondary (comorbid) diagnosis of dissocial personality disorder and seven (26%) presenting with a secondary diagnosis of emotionally unstable personality disorder. Therefore, more than 50% of our primary schizophrenia spectrum disorder patients presented with a comorbid personality disorder (either dissocial or emotionally unstable personality disorder).

Nineteen participants presented with a primary personality disorder diagnosis (39.6%), which was most commonly dissocial personality disorder.

Two participants presented with a pervasive developmental disorder (autism; 4.2%) and comorbid dissocial personality disorder and one participant presented with a primary bipolar affective disorder (2.1%). None of the participants had a formal diagnosis of ADHD. Patient’s index offence included sex offence, murder, manslaughter, grievous bodily harm (GBH), police/prison/court offence, gun/weapon offence, and repetitive violent assaults.

### Measures

*QbTest*—The QbTest (25) is an objective measure of inattention, impulsivity, and hyperactivity. The QbTest couples infrared motion tracking software with a CPT involving geometric shapes (squares or circles) of different colors (red or blue) appearing on a computer screen in a pseudo-random order. These images are each presented for 200 ms, with a 2-s interval between them. Participants are asked to press a responder button when two or more images that are identical in both color and shape appear one after the other. While they are performing the CPT, the participant’s movement is tracked by the infrared camera that detects a reflective ball worn on a headband. The adult version of the QbTest (age 12–60) lasts for 20 min, with the first 5 min excluded from the analysis to control for inconsistent responding patterns due to task adaptation. Data collected from the QbTest were stored *via* the software provided. Raw scores are transformed into Q-scores, corresponding to *z*-scores, after comparison to a normative database containing 1,307 age- and gender-matched controls published by Qbtech, Sweden. Q-scores greater than 1 indicate atypical performance (i.e., more inattentive, hyperactive, or impulsive than the normative population) with a Q-score of 1.6 corresponding to performance at the 95th percentile.

The QbTest consists of three domains, QbAttention, QbImpulsivity, and QbActivity. QbAttention and QbImpulsivity are measured by the CPT. QbActivity is measured during the course of the CPT *via* an infrared camera that tracks the path of a reflector attached to the participants head (central midpoint). QbAttention contains the main parameters of omission errors, reaction time, and reaction time variation, while QbImpulsivity contains the main parameters of commission errors and normalized commission errors. Although for each domain the main parameters are the most heavily weighted, the domains also contain information from each other’s parameters. For example, QbAttention is most weighted for omission errors, reaction time, and reaction time variation, but also contains information from commission errors and normalized commission errors. The different domains also take information from different parts of the test. The test is split into four quartiles, each 5 min in length. QbImpulsivity contains data from the second to fourth quartiles only, while QbAttention contains data from the third and fourth quartiles.

*IQ*—The Wechsler Test of Adult Reading (WTAR) (30) is a measure of premorbid intelligence, i.e., IQ before the onset of illness. It is thought to be a measure of “crystallized intelligence” as opposed to “fluid intelligence” (31). Patients were required to read aloud a list of 50 irregularly spelled words. They were scored on the accuracy of their pronunciation (correct/incorrect) and a total score out of 50 was obtained.

*The HCR-20*—The HCR-20 V3 is one of the most commonly used tools to assess risk (32, 33). It is a 20-item structured checklist of risk factors that have been linked to violent behavior, rated in terms of presence and relevance to the patient. The HCR-20 V3 is divided into three sections: “historical,” containing 10 items related to past risk factors such as previous substance use problems; “clinical,” containing 5 items related to current risk factors such as negative attitudes; and “risk management,” which consists of 5 items related to an individual’s capacity to cope in the future such as lack of personal support (34). While the historical total relates to static factors of risk, the clinical and risk management totals relate to dynamic factors of risk. Although for clinical purposes a total score is not generated, for research purposes it is deemed acceptable to add up the scores (absent, 0, partial, 1, or present, 2) to give an indication of total risk (35).

*Rehabilitative Engagement*—This was assessed from a structured review of the participant’s hospital records. All patients at the hospital undergo a progress review every 6 months with their multidisciplinary team, with individual reports produced by each discipline (e.g., psychology, occupational therapy, etc). It is therefore possible to gain a clinically informed impression of their current patterns of engagement with different therapeutic activities. For this study the level of engagement was recorded for 1) clinical therapies (1:1 nursing, individual, and group psychological therapy) and 2) vocational therapies engagement (occupational and vocational therapy such as working in the kitchen, attending woodwork courses, etc). rehabilitative activities. Engagement was rated on a five-point Likert scale: 0—complete refusal, 1—minimal, 2—intermittent, 3—regular, and 4—complete attendance.

### Design and Statistical Analysis

The study utilized a cross-sectional design. Quantitative data-analyses were conducted using IBM SPSS Statistics 22. For the first research question, one-sample *t*-tests compared inattention, impulsivity, and hyperactivity in this sample to the normative population. For the second and third research questions, Pearson’s correlations explored whether domains of inattention, impulsivity, and hyperactivity were associated with risk of violence or rehabilitative engagement. A hierarchical regression was run for the second research question in order to explore how much inattention contributed to the variance in occupational/vocational engagement. A hierarchical regression was then run for the third research question in order to explore how much omission errors contributed to the variance in the HCR-20 clinical and risk management total scores and occupational/vocational engagement. Age and IQ were controlled for within both analyses. All assumptions were met for these statistical tests.

### Procedure

After informed consent was obtained, an appointment was arranged for completion of the QbTest in private interview rooms on the hospital ward. Participants were shown a video that described the QbTest procedure. After completing the QbTest the participant was debriefed and given the opportunity to ask questions. The WTAR was completed on a previous occasion (within 1–6 months) as part of a battery of tests for another part of this research program. The researchers then completed a comprehensive file review in order to collect HCR-20 and the rehabilitative engagement data.

## Results

A one-sample *t*-test was conducted to determine if a statistically significant difference existed between inattention, impulsivity, and hyperactivity in MDOs compared to the normative population (*M* = 0, SD = 1). Results indicated that MDOs displayed significantly elevated levels of impulsivity [*t* (47) = 5.923, *p* < 0.001], inattention [*t* (47) = 8.723, *p* < 0.001], and hyperactivity [*t* (47) = 3.571, *p* < 0.001] relative to the general population (see **Table 1**).

**Table 1:** Observed t-values, degress of freedom, and statistical significance of the one-sample t-tests.

Domain	*df*	*t*	*p* <
Impulsivity	47	5.923	.001
Inattention	47	8.723	.001
Hyperactivity	47	3.571	.001

Pearson’s correlation analyses were run to explore the relation between the QbTest domains (QbActivity, QbImpulsivity, and QbInattention) risk of violence (HCR-20 historical total, HCR-20 clinical total, and HCR-20 risk management total) and rehabilitative engagement (clinical engagement and vocational engagement; see **Table 2**). Results revealed a significant negative correlation between the mean occupational/vocational engagement and QbInattention (*r* = −.352, *n* = 47, *p* < .005) suggesting that the more inattentive patients were, the less they engaged in occupational/vocational activities. All other correlations within the analysis were not statistically significant.

**Table 2:** Correlations between risk of violence, rehabilitative engagement, and the three Qb-test domains of inattention, impulsivity, and hyperactivity.

Risk measure	QbActivity	QbImpulsivity	QbInattention
HCR-20 Historical total	.108	−.057	.122
HCR-20 Clinical total	.261	−.018	.153
HCR-20 Risk-management total	.199	.142	.153
Clinical engagement	−.219	−.165	−.165
Vocational engagement	−.258	−.287	−.352*
Age	.251	−.165	−.043
IQ	−.229	−.052	−.099

*p < 0.05.

**Figure 1:**
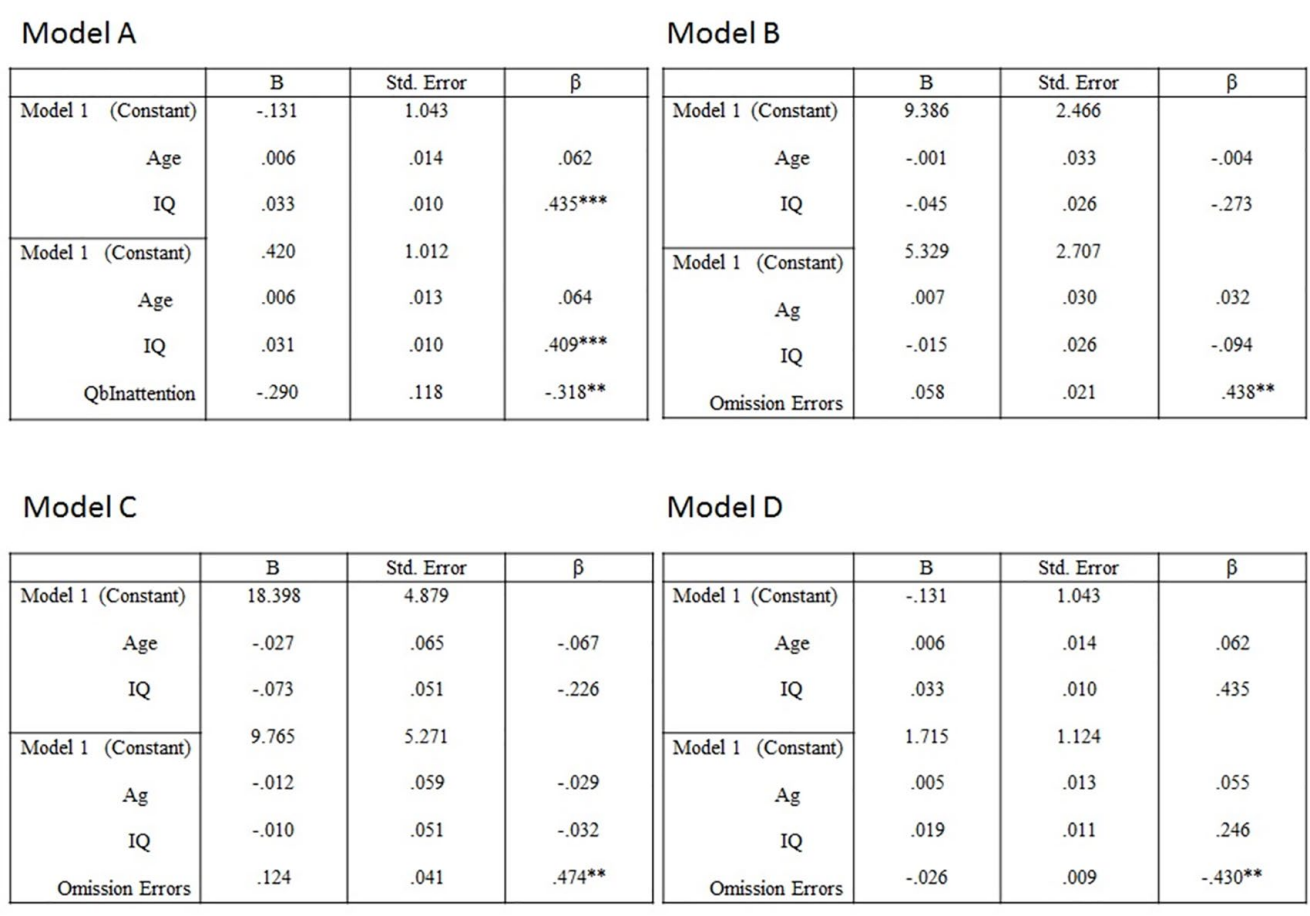
Hierarchical multiple regression models. **(A)** Vocational engagement (dependent variable); QbInattention (predictor), age and IQ (variables controlled for). R² = 0.16 for model; Δ R² = 0.10 for model 2 (ps < 0.01). *p < 0.05; **p < 0.01; ***p < 0.001. **(B)** Historical Clinical Risk Management-20 (HCR-20) clinical total (dependent variable), omission errors (predictor), age and IQ (variables controlled for). Note: R² = 0.07 for model; Δ R² = 0.15 for model 2 (p < 0.01). **(C)** HCR-20 risk management total (dependent variable), omission errors (predictor), age and IQ (variables controlled for). Note: R² = 0.06 for model; Δ R² = 0.18 for model 2 (p < 0.01). **(D)** Vocational engagement (dependent variable), omission errors (predictor), age and IQ (variables controlled for). Note: R² = 0.20 for model; Δ R² = 0.14 for model 2 (p < 0.01); Note *p < 0.05; **p < 0.01; ***p < 0.001.

A hierarchical regression (see **Figure 1**, model A) was run in order to explore how much variance in patients’ occupational/vocational engagement could be explained by inattention when age and IQ are controlled for. It was found that inattention significantly improved the prediction of the occupational/vocational engagement, Δ R² = 0.10 after controlling for age and IQ. Model coefficient indicated a negative association between inattention and occupational/vocational engagement, (standardized beta = −.318, *p* = <**0.01).

Following the same analysis procedure described above, Pearson’s correlation and hierarchical regressions were performed to explore whether omission and commission at the CPT task individually relate to indices of risk of violence or rehabilitative engagement. Pearson correlation results revealed significant positive correlations between omission errors and HCR-20 clinical total (*r* = .387, *n* = 47, *p* < .005) and risk management total (*r* = .473, *n* = 47, *p* < .001) scores. This suggests that the higher the number of omission errors, the higher the dynamic measures of risk of violence (see **Table 3**).

**Table 3:** Correlations between risk of violence, rehabilitative engagement, and continuous performance task (CPT) omission and commission errors.

Risk measure	Commision errors	Omission errors
HCR-20 Historical total	−.039	.253
HCR-20 Clinical total	−.026	.387*
HCR-20 Risk–management total	.163	.473**
Clinical engagement	−.253	−.350*
Vocational engagement	−.074	−.543**
Age	−.178	−.122
IQ	−.117	−.439**

*p < 0.05; **p < 0.01.

Omission errors also correlated significantly and negatively with clinical (*r* = −.350, *n* = 47, *p* < .005), occupational/vocational (*r* = −.543, *n* = 47, *p* < .001), engagement, and IQ (*r* = −.439, *n* = 47, *p* < .001) suggesting that the higher the number of omission errors, the lower the clinical, occupational/vocational engagement, and the IQ (see **Table 3**). All other correlations within the analysis were not statistically significant.

A series of hierarchical regressions were run to further explore how much variance in measures of risk of violence and rehabilitative engagement are explained by omission errors (indexing inattention). Model B (see **Figure 1**) shows that omission errors significantly improved the prediction of the HCR-20 clinical total, Δ R² = 0.15 after controlling for IQ and age. Model coefficient indicated a significantly positive association between omission errors and HCR-20 clinical total (standardized beta = 0.438, *p* < 0.01). Model C (see **Figure 1**) shows that omission errors significantly improved the prediction of the HCR-20 risk management total, Δ R² = 0.18 after controlling for IQ and age. Model coefficient indicated a significantly positive association between omission errors and HCR-20 risk management total, standardized beta = .474, *p* = <0.01. Model D (see **Figure 1**) revealed that omission errors significantly improved the prediction of occupational/vocational engagement, Δ R² = 0.14 after controlling for IQ and age. Model coefficient indicated a significantly negative association between omission errors and occupational/vocational engagement, standardized beta = −.430, *p* = <0.01. Omission errors did not significantly improve the prediction of clinical engagement.

## Discussion

The present study reports, for the first time, that when objectively assessed using the QbTest, MDOs detained in a high-security hospital displayed a significantly greater inattention, impulsivity, and hyperactivity than the general population, which is consistent with previous research (3, 36, 37). Although the QbTest was developed for use in the ADHD population, our results suggest that the QbTest is feasible to use in a forensic psychiatric population, is well tolerated by these patients, and provides useful outcomes.

The majority of participants in this study had either a primary schizophrenia spectrum or personality disorder diagnosis; this also fits with previous research that suggests that impulsivity and inattention are clinical features of these diagnoses (10, 38, 39). As previous research has utilized self-report measures, the current study builds on previous findings by using an objective form of assessing inattention and impulsivity.

The present study also explored whether the QbTest domains (inattention, impulsivity, and hyperactivity) and number of omissions and commission errors were related to risk of violence or rehabilitative engagement in MDOs. Results showed that only the domain of inattention was negatively related to occupational/vocational engagement, suggesting that the more inattentive the patients were the less they engaged in vocational and occupational activities. Inattention as measured by omission errors was also negatively associated with occupational/vocational engagement. These findings are consistent with a previous study that suggest that there is an association between inattention and disengagement in therapy (40). It is interesting that inattention predicted occupational/vocational engagement better than clinical engagement. Perhaps occupational/vocational activities place greater cognitive demand on patients than clinical activities involving attendance at individual and group sessions, which have a “talking therapy” focus. Furthermore, there may be motivational factors present with patients choosing to prioritize clinical over occupational/vocational activities because it is perceived as most likely to promote recovery and reduce length of stay.

In contrast to the overall lack of association between the inattention domain and risk of violence, inattention as measured by number of omission errors at the CPT task (part of the QbTest) was associated with the HCR-20 clinical and risk management total of present and future risk of violence.

Our findings are in keeping with previous studies in similar offender samples using different tasks but tapping on the attention domain. For example, our results are in keeping with Meijers et al. (21) who reported that violent offenders showed poor inhibition (stop-signal task) compared to nonviolent offenders. Our findings are also in accordance with other studies reporting attentional deficits in violent offenders with schizophrenia compared to nonviolent offenders with schizophrenia (20, 22). Our findings are also consistent with previous self-report studies that suggest that there is an association between inattention and assessed risk of violence (3, 40). The findings also suggest that inattention as defined by the self-control theory of crime may be an important factor for MDOs in leading to violent behavior. Therefore, these preliminary findings tentatively suggest that inattention is associated with risk of violence.

These findings are consistent with previous self-report observations in the literature, but how inattention may contribute to risk of violence in MDOs is not yet fully understood. This could be explained by the hostile attributional bias theory (41). This theory assumes that aggression arises due to multiple failures in information processing, including at the level of attentional encoding (42). When attention is limited, only partial cues are encoded, which can lead to erroneous interpretation of social cues and perceptions of hostile intent, which in turn can lead to violence (43). As such, according to this framework, the attention deficits in the current sample may contribute to impaired social understanding, and therefore risk of violence. Notably, associations with inattention were found for the HCR-20 clinical and risk management scores, which are the dynamic aspects of risk assessment, and not the historical score. This may also provide some insight as to the reason for less engagement in clinical activities as the clinical subscale considers active symptoms of mental illness such as hearing voices, which may impact on patients’ ability to concentrate in clinical sessions. Therefore, they may not engage fully as they gain fewer benefits from clinical interventions. Indeed, a stable mental state and attitude toward violence have been shown to be important predictors of clinical treatment dropout in this population (44). An important point that needs to be considered in the present study is the heterogeneity of our MDOs sample with regard to how this may have an effect on the results reported. It is important to highlight that about half of our sample has a primary diagnosis of schizophrenia spectrum disorder and the other half has a primary diagnosis of personality disorder (prevalently dissocial personality disorder). Indeed, the psychopathology and clinical course differ between the two categories in the general psychiatry population.

However, within the forensic psychiatry population (MDOs), patients perhaps display more complex clinical presentations. For example, in the present study more that 50% (15 out of 26) of our primary schizophrenia patients also had either comorbid dissocial or emotionally unstable personality disorder. Therefore, our sample included both primary schizophrenia spectrum and dissocial personality disorder patients. It is also important to consider that in the present study schizophrenia patients were stable (compliant to medication and free psychoses and significantly reduced positive symptoms) at the time of testing and mostly at lower levels of security (assertive rehabilitation wards) within the high-security hospital. A similar scenario applies to our dissocial PD who may have had psychosis episodes (and therefore referred to high security). They were stable (at the time of testing) and at a lower level of security (assertive rehabilitation wards) within the high-security hospital.

Overall, we suggest that, above and beyond their clinical diagnosis our sample of MDOs (schizophrenia and dissocial PD) are both very representative of the forensic psychiatry high-security population and overlap in terms of the overall risk of violent behavior toward others (and themselves) and their resistance to therapeutic engagement.

Another limitation of the present study is the lack of a control group with similar diagnoses as those of the present sample of MDOs but with no history of violent offending behavior. Had similar effects been found between MDOs and controls (non-violent) patients then this would have suggested a more robust link between attentional deficits and risk of violence and occupational/vocational engagement. Future research should be done to replicate the effects found in the present study employing a patient control group.

### Clinical Implications

Attention, impulsivity, and hyperactivity deficits in this sample of MDOs relative to normative scores suggest that their cognitive limitations need to be considered, particularly when devising risk management and treatment plans. Previous research has emphasized the need for MDOs to develop their cognitive skills by engaging in cognitive training (45). For example, the reasoning and rehabilitation program adapted for offenders with severe mental illness (R&R2MHP) is a cognitive skills program specifically adapted for MDOs, which addresses antisocial and offending behavior by helping offenders to develop their cognitive and social skills, such as self-control (46). Research has indicated that R&R2MHP is an effective treatment for MDOs detained in low-, medium-, and high-security hospitals, and is associated with a low dropout rate and significant improvements in self-reported violent attitudes, coping processes, social problem solving, and cognitive improvements (46, 47). The current findings emphasize the need for forensic services to provide cognitive skills programs that specifically include modules to address deficits in attention and impulsivity and that have demonstrated low dropout rates.

Tentatively, the findings suggest that improvements in attention may contribute to a reduction of risk of present and future violence. However, this research is correlational and there may be other factors not assessed here that may be influential. Inattention was strongly related to risk of present and future violence; hence, it may be possible to reduce this risk and/or monitor risk reduction by the introduction of clinical programs to address the development of cognitive skills. However, further investigation is needed in order to establish a clear, causal link between inattention and its prediction of risk of present and future violence in MDOs, particularly before establishing changes in clinical practices.

## Conclusion

This study demonstrated significantly elevated level of inattention, impulsivity, and hyperactivity in a forensic sample with a history of severe mental illness. Of these, inattention emerged as a strong predictor of patient’s risk of violence as well as patient’s vocational/occupational therapy engagement. The present findings suggest that improved attention would lead to better engagement and a more favorable clinical outcomes in this population. Finally the present study demonstrates that QbTest is a helpful objective tool that could beneficially be incorporated into the routine assessment of MDOs.

## Ethics Statement

The authors assert that all procedures contributing to this work comply with the ethical standards of the relevant national and institutional committees on human experimentation and with the Helsinki Declaration of 1975, as revised in 2008. The study was reviewed and approved by the National Research Ethics Service (14/LO/0238) and the West London Mental Health Trust Research and Development (96463/LNW).

## Author Contributions

IP, SY, and OS contributed to the formulation of the research questions. IP, RK, and BG contributed to data collection. IP, OS, RK, and BG preprocessed the data. IP, VK, and GG analyzed the data. IP, OS, VK, GG, and SY interpreted the results and drafted, critically revised, and approved the version to be published. RK and BG drafted and approved the version to be published.

## Conflict of Interest Statement

The authors declare that the research was conducted in the absence of any commercial or financial relationships that could be construed as a potential conflict of interest.
